# A pilot study of a new test to predict extubation failure

**DOI:** 10.1186/cc7783

**Published:** 2009-04-14

**Authors:** José F Solsona, Yolanda Díaz, Antonia Vázquez, Maria Pilar Gracia, Ana Zapatero, Jaume Marrugat

**Affiliations:** 1 ICU Hospital de Mar, Paseo Maritimo 25-29 Barcelona 08003, Spain; 2 Institut Municipal d'Investigacio Medica, C/Aiguader 80, Barcelona 08003, Spain

## Abstract

**Introduction:**

To determine whether subjecting patients to 100 ml of additional dead space after a 120-minute weaning trial could predict readiness for extubation.

**Methods:**

This was a prospective, non-randomised pilot study in an intensive care unit at a university hospital with 14 beds. It included all non-tracheostomised patients with improvement of the underlying cause of acute respiratory failure, and those with no need for vasoactive or sedative drugs were eligible. Patients fulfilling the Consensus Conference on Weaning extubation criteria after 120 minutes spontaneous breathing (n = 152) were included. To the endotracheal tube, 100 cc dead space was added for 30 minutes. Patients tolerating the test were extubated; those not tolerating it received six hours of supplementary ventilation before extubation. The measurements taken and main results were: arterial pressure, heart rate, respiratory rate, oxygen saturation, end-tidal carbon dioxide and signs of respiratory insufficiency were recorded every five minutes; and arterial blood gases were measured at the beginning and end of the test. Extubation failure was defined as the need for mechanical and non-invasive ventilation within 48 hours of extubation.

**Results:**

Twenty-two patients (14.5%) experienced extubation failure. Only intercostal retraction was independently associated with extubation failure. The sensitivity (40.9%) and specificity (97.7%) yield a probability of extubation failure of 75.1% for patients not tolerating the test versus 9.3% for those tolerating it.

**Conclusions:**

Observing intercostal retraction after adding dead space may help detect susceptibility to extubation failure. The ideal amount of dead space remains to be determined.

**Trial registration:**

Current Controlled Trials ISRCTN76206152.

## Introduction

Mechanical ventilation is a life-maintaining intervention; however, it may be associated with unwanted side effects and life-threatening complications [1] and should thus be discontinued as soon as possible.

For this reason, diverse methods to predict the success or failure of weaning have been evaluated [2-11]. The American College of Chest Physicians recommends periodic weaning trials consisting of brief periods of spontaneous breathing in which the respiratory pattern, gas exchange, haemodynamic stability and patient comfort are evaluated.

Nevertheless, between 12 and 25% of patients extubated after successful weaning trials experience post-extubation respiratory insufficiency and require reintubation [9,10]. The patients that require reintubation are apparently indistinguishable from those who are successfully extubated. In part, this is because extubation failure is often caused by factors different from those that cause failure in weaning trials [12]. Several studies have identified patients for extubation and have shown that factors such as neurological status [13,14], cough strength [13-15] and amount of endotracheal secretions [15] may be important predictors of extubation outcomes. The most common reasons for reintubation are airway obstruction and the inability to eliminate secretions. As reintubation is clearly associated with a worsened prognosis, decreasing the extubation failure rate is important [16].

To date, the ability to tolerate 30 to 120 minutes of spontaneous breathing has generally been considered the gold standard for identifying patients that are ready for extubation. We hypothesise that it is possible to further identify patients that are likely to require reintubation by subjecting patients to a burden in addition to that supposed by the spontaneous breathing trial. The response to this burden could facilitate data that might be useful in deciding whether to extubate and help to reduce extubation failure. To this end, we carried out a study in which an additional burden of 100 cc dead space was added to the endotracheal tube after 120 minutes of successfully tolerated spontaneous breathing.

This study aimed to determine the clinical and gasometric parameters registered during the additional burden breathing trial that are most reliable in predicting extubation failure. Some of the results of this study have previously been reported in abstract form [17].

## Materials and methods

This is a prospective, non-randomised pilot study of the dead space addition (DSA) test which aims to detect increased risk of extubation failure. The study was carried out between November 2004 and October 2005 in a 14-bed intensive care unit (ICU) at a university hospital in Barcelona. The institution's ethics and clinical trials committee approved the study, and informed consent was obtained from all participating patients or from their relatives.

### Inclusion criteria

Included in the study were consecutive patients who tolerated a spontaneous T-piece breathing trial of 120 minutes initiated in patients that fulfilled all of the following criteria: improvement of the underlying cause of acute respiratory failure; adequate gas exchange characterised by a partial pressure of arterial oxygen (PaO_2_) more than 60 mmHg with fraction of inspired oxygen (FiO_2_) of 0.4 or less with positive end-expiratory pressure (PEEP) of 5 cmH_2_O or less; Glasgow Coma Score of more than 13; body temperature of 38°C or below; and no need for vasoactive or sedative drugs. Tracheostomised patients were excluded. The DSA test was only performed in patients that fulfilled the following criteria for extubation recommended by the Consensus Conference on Weaning after the 120-minute spontaneous breathing trial: no signs of respiratory insufficiency (paradoxical breathing, abdominal breathing, agitation, excessive sweating, etc); pulse oximetry more than 90% with FiO_2 _less than 0.5; respiratory rate (RR) less than 35 breaths/minute; and less than 20% variation in heart rate (HR) and blood pressure (BP).

The following data were recorded for all patients: simplified acute physiology score (SAPS) II at ICU admission, number of days on mechanical ventilation, the presence of chronic obstructive pulmonary disease (COPD), demographic and anthropometric variables.

### Dead space addition test procedure

**Figure 1:**
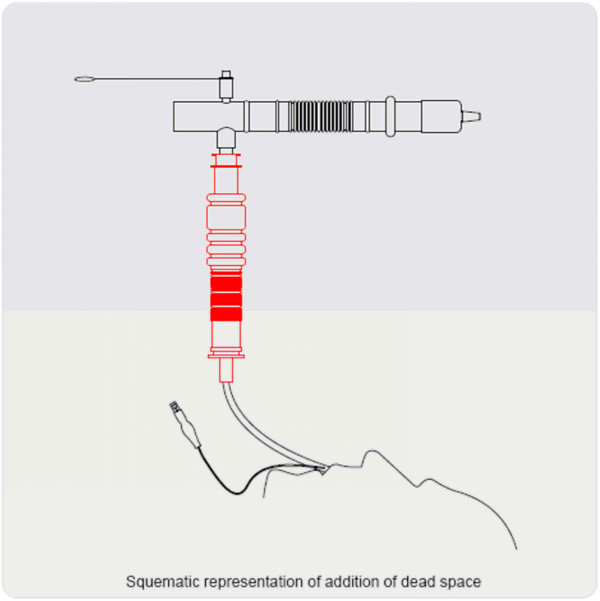
Schematic representation of the addition of dead space.

The DSA test consisted of adding a tube with an internal volume of 100 cc (measured by filling the tube with water) between the endotracheal tube and the T-piece with oxygen for 30 minutes (Figure 1). This test was performed in all patients that fulfilled the criteria for extubation. At the start of the test, BP, HR, RR, oxygen saturation by pulse oximetry and end-tidal carbon dioxide were measured. These measurements were repeated every five minutes. The attending physician and researcher were at the patient's bedside throughout the test.

Clinical signs such as intercostal retractions, accessory muscle use and nasal flaring were monitored in all patients. The use of accessory muscles was defined as the contraction of the sternomastoid muscles. Intercostal retraction was defined as indrawing of the intercostal space during inspiration [18]. Nasal flaring was defined as active flaring of the nostrils. Arterial blood gases were recorded at the beginning and end of the test for further analysis.

Successful toleration of the DSA test was determined using the Consensus Conference on Weaning criteria described above. Patients that successfully tolerated the test were extubated immediately after this 30-minute period of spontaneous ventilation with DSA test.

Prior to extubation of patients we ensured the cough capacity was correct and volume of secretions was not excessive.

Whenever patients failed to tolerate the test, it was interrupted immediately. All patients that failed to tolerate the test received six hours of assist-control ventilation to aid in recovery from possible respiratory fatigue. After this recovery period, patients underwent a new 120-minute T-piece spontaneous breathing trial prior to extubation. Extubation failure was defined as the need for reintubation or non-invasive mechanical ventilation within 48 hours of extubation.

The causes for failure after extubation were classified according to Epstein and colleagues [19] on respiratory failure, congestive heart failure, aspiration or excess secretions, upper airway obstruction or encephalopathy.

The researcher was not involved in the decision to reintubate or apply non-invasive mechanical ventilation. Mechanical or non-invasive ventilation was applied according to the Consensus Conference on Weaning [20].

### Statistical analysis

The results for the continuous variables are presented as mean and standard deviation or median and interquartile interval if they did not fit a normal distribution. The comparisons of the mean/median values between the groups of extubation results, and of DSA result, were made using Student's *t*-test or the Mann-Whitney U test depending on the whether the distribution departed from the normal. The chi-squared test was used to compare proportions for categorical variables between groups.

To determine the best criteria for 30-minute DSA test failure we used a non-conditional logistic regression model. The mutually adjusted odds ratio (OR) of extubation failure was estimated in a model with the variables that were significantly (*P *< 0.05) associated in the bivariate analysis.

The *a posteriori *probability of success or failure of the 30-minute DSA test was calculated using Bayes' theorem, taking the extubation failure rate using the classical T-tube test method (14.5% in our centre, which is similar to that found in the present study) to be the *a priori *probability.

The formula for calculating the *a posteriori *probability requires the transformation of the pre-test probability to an odds [odds = probability/(1 - probability)].

For a failed test, a higher risk of extubation failure detected, that is post-test odds (failed test) = pre-test odds × (sensitivity/(1 - specificity)).

For a successful test, that is post-test odds (successful test) = pre-test odds × ((1 - sensitivity)/specificity).

To convert the post-test odds to a percentage, the following calculation was performed: probability = (odds/(1+odds)).

## Results

A total of 152 patients passed the 120-minute T-piece spontaneous breathing trial and were thus included in the study. Twenty-two patients required invasive or non-invasive ventilation within 48 hours of extubation (extubation failure 14.5%).

Table 1 shows the characteristics of the patients that were successfully extubated and those that experienced extubation failure. Statistically significant differences were found between the two groups for days on mechanical ventilation, and for the increments observed in mean RR and increased intercostal retraction, and marginally significant for age during the DSA test.

**Table 1:** Characteristics of patients according to extubation success or failure, as well as the parameters measured at the start of the dead-space test and the increase occurring during the test

	Extubation failure		Successful extubation		
	n = 22		n = 130		
			
	Mean	(SD)	Mean	(SD)	*P *value
GENERAL DATA					
Age (years)	66	(14)	59	(17)	0.070
Women (%)	10 (47%)		35 (27%)		0.103
COPD (%)	6 (27%)		47 (36%)		0.477
SAPS II	38	11	35	14	0.508
Days under mechanical ventilation: days (median and interquartile range)	11	(9.5 to 34)	6	(3 to 11)	0.010
BEFORE THE TEST					
Mean blood pressure	95	(12)	93	(13)	0.406
Heart rate (beats/minute)	91	(14)	88	(17)	0.548
Respiratory rate	24	(6)	24	(9)	0.873
PaO_2 _(FiO_2 _0.4; mmHg)	94	(35)	99	(43)	0.611
pCO_2 _(mmHg)	44	(12)	42	(10)	0.307
End-tidal carbon dioxide (mmHg)	36	(9)	34	(8)	0.227
(PCO_2 _– end-tidal carbon dioxide) gradient	7	(9)	7	(9)	0.925
DURING THE TEST					
Increase in mean blood pressure (mmHg)	6	(17)	0	(9)	0.131
Increase in heart rate (beats per minute)	11	(24)	3	(11)	0.146
Increase in respiratory rate	7	(9)	1	(7)	0.001
Decrease in PO_2 _(mmHg)	0	(41)	3	(34)	0.650
Increase in PCO_2 _(mmHg)	7	(19)	1	(5)	0.178
Increase in end-tidal carbon dioxide (mmHg)	1	(6)	1	(6)	0.982
Increase in PCO_2_-end-tidal carbon dioxide gradient (mmHg)	3	(10)	-0.2	(8)	0.150
Increased work of breathing (intercostal retraction)	9 (41%)		3 (2%)		< 0.001

COPD = chronic obstructive pulmonary disease; FiO_2 _= fraction of inspired oxygen; PaO_2 _= partial pressure of arterial oxygen; pCO_2 _= partial pressure of carbon dioxide; SAPS = simplified acute physiology score; SD = standard deviation.

Table 2 shows the association between the variables measured (worst quartile of the difference between basal and final values vs. the rest of quartiles) during the test and extubation failure. In the logistic regression model with the variables measured mutually adjusted, the only variable independently associated with extubation failure was intercostal retraction during the test.

**Table 2:** Mutually adjusted odds ratio of extubation failure for the worst quartile of the differences between the start and end of the test for the parameters*

	Odds ratio	95% confidence interval	
Difference in mean blood pressure	2.67	0.30	23.93
Difference in heart rate	1.2	0.37	6.31
Difference in respiratory rate	0.62	0.16	2.41
Difference in PCO_2_	0.66	0.15	2.85
Age (one year)	1.02	0.98	1.06
Increased work of breathing (intercostal retraction)	56.67	3.55	905.41

* This was measured in comparison to the rest of the quartiles (up to interruption in failed tests) and increased the work of breathing during the test.

pCO_2 _= partial pressure of carbon dioxide.

Table 3 shows the sensitivity, specificity and predictive values for test failure as a predictor of extubation failure when increased work of breathing is the only variable taken into account. Twelve patients failed the DSA test: the test was interrupted in six patients and the other six no longer met the Consensus Conference for Weaning criteria for extubation at the end of the test. Extubation failure occurred in nine of the 12 patients that failed the test; six required reintubation and the remaining three requiring non-invasive mechanical ventilation. The DSA test detected the cases of extubation failure due to respiratory failure (eight of nine) and aspiration excess secretion (one of nine).

**Table 3:** Characteristics of the added dead-space test for predicting the outcome of extubation, considering clinical signs of respiratory insufficiency

	Extubation failure	Successful extubation	Total		
Increased work of breathing (failed dead-space test)	9	3	12	Positive predictive value	75.0%
No increase in work of breathing (not failed dead-space test)	13	127	140	Negative predictive value	90.7%
	22	130	152		
	**Sensitivity**	**Specificity**			
	40.9%	97.7%			
Likelihood ratio for a positive test	17.8				

Sensitivity represents the proportion of true positive tests, which is the proportion of patients with increased work of breathing in whom extubation failed.

**Figure 2:**
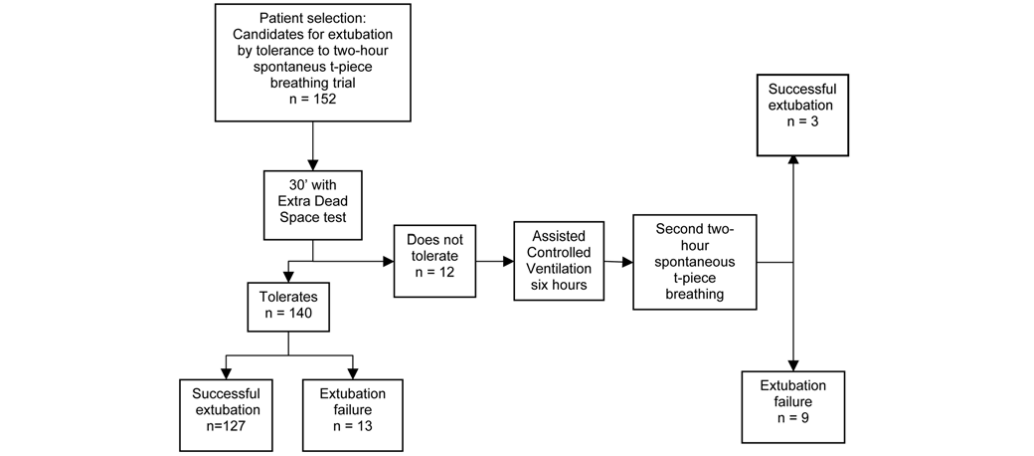
The evolution of the patients.

Extubation failure occurred in 13 of the 140 patients that successfully tolerated the test; 10 of these patients were reintubated and three required non-invasive ventilation (Figure 2).

Table 4 shows the characteristics of the patients that failed the DSA test (intercostal retraction) and those that passed it. Patients failing the test were significantly older, had higher partial pressure of carbon dioxide (pCO_2_) at the start of the test, and larger increases in mean BP, HR and RR during the test. All patients in whom the test was interrupted presented with intercostal retraction as the first sign of increased work of breathing. No other signs of increased work of breathing (accessory muscle use, nasal flaring) were detected, because the patient was connected to the mechanical ventilator at the slightest sign of increased respiratory work.

**Table 4:** Characteristics of the patients by increased work of breathing, as well as the response of the variables to the dead-space addition test

	Positive: With increased work of breathing		Negative: Without increased work of breathing		
	n = 12		n = 140		
			
	Mean	(SD)	Mean	(SD)	*P *value
Age (years)	71.5	(13.4)	59.2	(17.0)	0.027
Extubation failure (%)	75.0%		9.3%		< 0.001
Women (%)	50.0%		21.1%		0.144
COPD (%)	33.3%		35.0%		1.000
SAPS II	39	(14)	35	(13)	0.403
Days on mechanical ventilation: days (median and interquartile range)	6	(3 to 11)	9	(5 to 17.5)	0.193
Mean blood pressure (mmHg)	97	(14)	93	(13)	0.241
Heart rate (beats/minute)	92	(12)	88	(17)	0.476
Respiratory rate	23	(6)	24	(9)	0.855
PaO2 (mmHg)	88	(33)	99	(42)	0.440
pCO2 (mmHg)	51	(13)	41	(10)	0.363
End-tidal carbon dioxide (mmHg)	37	(8)	34	(8)	0.396
PCO2 – end-tidal carbon dioxide gradient (mmHg)	14	(12)	7	(9)	0.665
Increase in mean blood pressure (mmHg)	20	(12)	0	(9)	< 0.001
Increase in heart rate (beats/minute)	24	(29)	2	(10)	0.027
Increase in respiratory rate	13	(11)	1	(6)	0.040
Decrease in PaO2 (mmHg)	-14	(26)	4	(35)	0.178
Increase in pCO2 (mmHg)	6	(12)	1	(8)	0.156
Increase in end-tidal carbon dioxide (mmHg)	4	(7)	1	(6)	0.086
Increase in pCO2 – end-tidal carbon dioxide gradient (mmHg)	2	(15)	0	(8)	0.782

COPD = chronic obstructive pulmonary disease; PaO_2 _= partial pressure of arterial oxygen; pCO_2 _= partial pressure of carbon dioxide; SAPS = simplified acute physiology score; SD = standard deviation.

Taking into account the sensitivity and specificity of the DSA test (40.9% and 97.7%, respectively, which leads to a likelihood ratio of 17.8), the probability of extubation failure (calculated according to Bayes' theorem) after a failed test would be 75.1% versus 9.3% after a successful test.

## Discussion

Our results suggest that the addition of 100 cc of dead space to the endotracheal tube for 30 minutes in candidates for extubation that have successfully passed a spontaneous breathing trial of 120 minutes can identify a subgroup of patients with increased risk of extubation failure. Clinical observation of increased work of breathing, which in our patients was always expressed by intercostal retraction, has proven particularly important, being the only variable that was independently associated with extubation failure (Table 2). This finding is compatible with those reported by Cham and colleagues [18], who identified intercostal retraction as an early clinical sign of respiratory insufficiency in the exacerbation of patients' asthma or COPD. Likewise, using electromyography, Duiverman and colleagues [21] identified an increase in the activity of the intercostal muscles an early sign of respiratory failure in COPD patients breathing against an inspiratory load. We used the same definition of intercostal retraction, that is the indrawing of the intercostal spaces during respiration, as Cham and colleagues [18]. This clinical observation is easily detected at the bedside and as such can be standardised. The difference between the measurements of mean BP, HR, RR and PaO_2 _at the beginning of the test and the values measured every five minutes did not improve the prediction. In our opinion, this is because the patient was carefully supervised throughout the test and immediately connected to the mechanical ventilator at the first signs of respiratory failure; thus, severe deterioration of gas exchange was not allowed to occur.

The originality of this study lies in the fact that it is the first to use a stress test to determine the likelihood of extubation failure. Being able to tolerate an additional workload might, in theory, demonstrate the capacity of respiratory musculature reserves and the capacity to maintain greater breathing efforts for a longer time.

The patients that were unable to tolerate the additional dead space had an extubation failure rate of 75%. These patients had been mechanically ventilated for a mean of 16 days and had higher levels of pCO_2 _at the start of the test than those that tolerated the additional workload. On the other hand, on average the patients that did not tolerate the test had increased BP, HR and RR. However, in the logistic regression analysis, intercostal retraction was the only parameter that was significantly associated with extubation failure owing to the fact that severe deterioration of gas exchange was not allowed to occur.

As this is a pilot study, the choice of the additional dead-space burden was tentative, although the idea was to reproduce in part the physiological situation that the patient would undergo once extubated. The anatomic dead space comprised in the upper airways and the intrathoracic airways is approximately 2 ml/kg, that is about 150 ml in a normal adult [22]. A study in cadavers found a mean extrathoracic dead space of approximately 75 ml [23], which is clearly greater than the dead space contained in endotracheal tubes. Davis and colleagues [24] measured the dead space in several different endotracheal tubes at approximately 24 ml for an 8.5 mm tube. Therefore, on withdrawal of the endotracheal tube, the average patient's dead space increased by approximately 50 ml (75 to 24 ml). Thus, considering the increase of 50 ml the patient would face at extubation, we arbitrarily chose to add another 50 ml of dead space in an attempt to ensure an effective burden without overtaxing the patient's respiratory system.

We opted for a fixed volume of added dead space as it is impossible to measure the individual variation in dead space occurring at extubation, because the patient is intubated. Obviously, this means that there might very well be small differences among patients (probably related to body surface area); however, we believe that the method gains in simplicity, ease of application and cost containment for bedside use.

With the chosen challenge, which was arbitrarily selected, the positive predictive value of test failure for extubation failure was 75%, that is three patients did not tolerate the test but did not experience extubation failure. Therefore, it seems that the burden applied was greater than that necessary for spontaneous respiration after extubation. We believe that a lesser burden (70 ml) could reduce the percentage of false positives, although it is highly unlikely that any predictive test can reach 100% specificity.

However, 13 patients tolerated the DSA test but nevertheless experienced extubation failure (Table 3). Extubation failure has numerous causes, including airway obstruction, inadequate cough, an excess of secretions and cardiac dysfunction [14,25]; thus, at best we could only decrease it by a relative percentage of the total. Indeed, the DSA test cannot detect glottal oedema or acute pulmonary oedema due to left ventricular failure; therefore, other tests would be necessary to enable more reliable prediction of extubation failure.

As is shown in Table 1, the study population consisted of medical and surgical patients with high SAPS II scores that had remained on mechanical ventilation for a long time. The extubation failure rate of our series (14.5%) is similar to that observed in other studies [9,10]. However, due to the small sample size, unlike other series [26], the proportion of COPD patients in the extubation failure group was not greater than in successfully extubated patients (Table 4).

The patients that failed the DSA test were characterised by signs of intercostal retraction in response to the added burden and were connected to the mechanical ventilator in assist-control mode for six hours. This procedure was intended to ensure that the DSA test itself did not induce extubation failure due to muscle fatigue. As these patients were immediately connected to the mechanical ventilator at the first clinical sign of respiratory work and were not allowed to finish the test, it is unlikely that fatigue developed. Moreover, all of these patients went on to pass a new 120-minute spontaneous breathing trial before extubation. Laghi and colleagues [27] found that none of the 11 patients in their study that failed the weaning trial developed low-frequency fatigue. Finally, studies [28,29] indicate that clinically significant respiratory muscle fatigue rarely occurs during well-monitored weaning trials, and that even if fatigue should develop, recovery may be rapid. Therefore, we consider six hours' mechanical ventilation to be an adequate compromise between reversing the improbable fatigue and preventing ventilator-induced diaphragmatic dysfunction [30].

There are analyses that show that reintubation can be independently associated to severity-adjusted mortality [16], and there has been a growing interest in predicting extubation failure [31-33]. To date, only the application of non-invasive ventilation in selected patients has proven useful in preventing reintubation [34,35]. However, a large multicentre study in patients similar to ours concluded that the application of noninvasive ventilation in patients with extubation failure failed to reduce reintubation or mortality rates [36].

If our hypothesis is confirmed, the non-invasive ventilation could be applied to those patients who are at risk of extubation failure, that is to say, in those who experience an increase of intercostal retraction during the DSA test.

The limitations of this study derive fundamentally from the idea on which it is based. In effect, the practice of subjecting patients to an added respiratory burden to try to improve the sensitivity and specificity of the tests to judge patient readiness for extubation generates doubt as to whether the added burden itself might have caused extubation failure in some patients who would not have failed otherwise. Nevertheless, there are three arguments against this hypothesis. First of all, any deleterious effects of the test itself would be expected to be increase the extubation failure rate of this series, and we found no difference with the rate observed in all patients admitted to our ICU in the five years before the study. Second, the test was discontinued immediately at the first sign of respiratory insufficiency and these patients were then mechanically ventilated for six hours; all of these patients went on to tolerate a new 120-minute weaning trial; thus, it is unlikely that they would have developed muscle fatigue. Third, three patients did not tolerate the added burden but did not experience extubation failure.

In some patients, 100 ml of dead space may constitute too large a ventilatory load – essentially precipitating failure (and possible respiratory muscle fatigue) in patients who would have otherwise tolerated weaning. If this were to occur, the additional six hours of mechanical ventilaton, prior to extubation, would be insufficient to rest the muscles [37]. This concern is relevant as women constitute nearly half of the extubation failures.

Another limitation of using intercostal retraction is the subjective nature of the finding, particulary in obese patients (in our series 0 of 12). Although it is of a subjective nature, we followed the clinical criteria described by Cham and colleagues [18].

Finally given the design of the study it was not possible to blind those making decisions about extubation and reintubation. Blinding was not possible because all who failed the DSA were placed back on mechanical ventilation and given another spontaneous breathing test.

## Conclusions

If the usefulness of the DSA test were confirmed in larger studies, this test may help identify a substantial proportion of patients that will experience extubation failure and thereby reduce extubation failure and mortality rates in critical patients. Simply observing intercostal retraction after adding dead space may help detect susceptibility to extubation failure, although the ideal amount of dead space remains to be determined.

## Key messages

• The addition of 100 cc of dead space to the endotracheal tube for 30 minutes in candidates for extubation can identify a subgroup of patients with increased risk of extubation failure.

• Clinical observation of increased work of breathing, which in our patients was always expressed by intercostal retraction, has proven particularly important, being the only variable that was independently associated with extubation failure.

• The originality of this study lies in the fact that it is the first to use a stress test to determine the likelihood of extubation failure.

• The patients that were unable to tolerate the additional dead space had an extubation failure rate of 75%.

• As this is a pilot study, the choice of the additional dead-space burden was tentative, although the idea was to reproduce in part the physiological situation that the patient would undergo once extubated.

## Abbreviations

BP: blood presssure; COPD: chronic obstructive pulmonary disease; DSA: dead space addition; FiO_2_: fraction of inspired oxygen; HR: heart rate; ICU: intensive care unit; OR: odds ratio; PaO_2_: partial pressure of arterial oxygen; pCO_2_: partial pressure of carbon dioxide; PEEP: positive end-expiratory pressure; RR: respiratory rate; SAPS: simplified acute physiology score.

## Competing interests

The authors declare that they have no competing interests.

## Authors' contributions

JFS and AV participated in the study design. YD and MPG performed the DSA test. YD, MPG and AZ performed the acquisition of data. JM performed the statistical analysis and contributed to the study design. JFS, AV, YD and MPG performed the interpretation of the data and helped to draft the manuscript. All authors read, edited and ultimately approved the final manuscript.
